# Low concentration flufenamic acid enhances osteogenic differentiation of mesenchymal stem cells and suppresses bone loss by inhibition of the NF-κB signaling pathway

**DOI:** 10.1186/s13287-019-1321-y

**Published:** 2019-07-19

**Authors:** Xuenan Liu, Zheng Li, Hao Liu, Yuan Zhu, Dandan Xia, Siyi Wang, Ranli Gu, Weiliang Wu, Ping Zhang, Yunsong Liu, Yongsheng Zhou

**Affiliations:** 1 Department of Prosthodontics, Peking University School and Hospital of Stomatology, National Laboratory for Digital and Material Technology of Stomatology, Beijing Key Laboratory of Digital Stomatology, National Clinical Research Center for Oral Diseases, 22 Zhongguancun South Avenue, Beijing, 100081 People’s Republic of China; 2 Department of Materials Science and Engineering, College of Engineering, Peking University, Beijing, 100871 People’s Republic of China; 3 Department of Implantology II, The Affiliated Stomatological Hospital of Fujian Medical University, Fuzhou, 350001 Fujian People’s Republic of China

**Keywords:** Flufenamic acid, Mesenchymal stem cells, Osteogenesis, Osteoporosis, Nuclear factor-κB

## Abstract

**Background:**

As the representative of fenamic acids, an important group of NSAIDs, flufenamic acid (FFA) has been used for anti-inflammation and analgesia in the clinic. Recently, researches have focused on the role of some members of NSAIDs in promoting osteogenesis. However, little attention has been paid to the subgroup of fenamic acids, and it remains unclear whether FFA and other fenamic acids could regulate mesenchymal stem cells’ (MSCs) lineage commitment and bone regeneration.

**Methods:**

Here we treated two kinds of human MSCs with FFA at different concentrations in vitro and examined the effect of FFA on osteogenic differentiation of human MSCs. This was followed by heterotopic bone formation assay in nude mice. In addition, ovariectomized and aged mice were used as osteoporotic models to test the effect of FFA on osteoporosis. Besides, activators and inhibitor of nuclear factor-κB (NF-κB) signaling pathway and western blot were used to clarify the mechanism of the promoting effect of low concentration FFA on osteogenesis.

**Results:**

Our results indicated that low concentrations of FFA could significantly enhance osteogenic differentiation of human MSCs in vitro, as well as in vivo. In addition, FFA treatment suppressed bone loss in ovariectomized and aged mice. Mechanistically, FFA at low concentrations promoted osteogenesis differentiation of human MSCs by inhibition of the NF-κB signaling pathway.

**Conclusions:**

Collectively, our study suggested that low concentration FFA could be used in bone tissue engineering or osteoporosis by promoting osteogenic differentiation of human MSCs.

**Electronic supplementary material:**

The online version of this article (10.1186/s13287-019-1321-y) contains supplementary material, which is available to authorized users.

## Background

As a common disease that can often be seen in the clinic, osteoporosis brings health and economic burdens to patients and remains a clinical challenge. It is usually caused by estrogen deficiency in menopausal women or by aging in men [1], leading to low bone strength, caused by low mineral density, architectural deterioration of the bone structure, and susceptibility to fracture [2]. Previous studies have shown that abnormal in osteogenic differentiation capacity of bone marrow-derived mesenchymal stem cells (BMMSCs) contributes to these structural abnormalities [3–5], which is mainly caused by loss of bone homeostasis.

Non-steroidal anti-inflammatory drugs (NSAIDs) are commonly used in the clinic and can inhibit the synthesis of prostaglandin by inhibiting cyclooxygenases (COXs) to achieve the anti-inflammatory and analgesia effects [6–8]. However, the members of the NSAIDs are quite different in their chemical structures and other properties, which may cause different effects in addition to anti-inflammatory and analgesia effects. According to their chemical structure, NSAIDs can be divided into several groups, for instance, salicylic acids, anilines, acetic acids, oxicams, and fenamic acids [9]. Some members of NSAIDs have been proven to have complex effects on the biological behaviors of mesenchymal stem cells (MSCs) and bone homoeostasis, such as aspirin [10], ibuprofen, and indomethacin [11]. However, little attention has been paid to the important group of fenamic acids. In particular, the effect of fenamic acids on MSC fate has not been reported.

A variety of transcription and signaling factors participate in lineage commitment of MSCs toward osteogenic cells, including the nuclear factor kappa B (NF-κB) signaling pathway. As a regulator of immune and inflammatory signals [12, 13], the NF-κB signaling pathway plays an important role in regulating the differentiation and function of osteocytes and in bone metabolism [14–18]. Activation of this pathway can prevent osteogenic differentiation of MSCs [14] and inhibit osteoblastic SMAD activation [18] and the synthesis of matrix protein in bone [19], as well as suppressing postnatal bone formation in vivo [15]. Besides, several genes participate in bone metabolism through the NF-κB signaling pathway [20, 21]. However, whether fenamic acids can affect the functions of MSCs through the NF-κB signaling pathway remains unknown.

In the present study, we aimed to explore the in vitro and in vivo effects of FFA, a representative of the fenamic acids of NSAIDs, on the osteogenic differentiation of hMSCs and its potential mechanism. The in vivo effects of FFA in an osteoporotic animal model were also involved. Our results demonstrated that low concentration FFA promoted osteogenic differentiation of hMSCs in vitro and the effect declined with the concentration increasing. The best concentration for osteogenesis promoting was 50 μM. Fifty micromolar FFA could also promote osteogenic differentiation of hMSCs in vivo. Besides, 50 μM FFA suppressed bone loss in OVX mice and aged mice. Mechanistically, FFA promoted osteogenesis through inhibition of NF-κB signaling pathway. Overall, our study provided valuable clues for the potential use of FFA to treat bone metabolic disease and in bone tissue engineering.

## Methods

### Culture and osteogenic induction of hMSCs

Primary human BMMSCs and human adipose-derived mesenchymal stem cells (hASCs) were purchased from ScienCell Company (San Diego, CA, USA). All cell-based in vitro studies were repeated three times using MSCs from three donors. All materials were purchased from Sigma-Aldrich (St. Louis, MO, USA) unless otherwise stated. This study was approved by the Institutional Animal Care and Use Committee of the Peking University Health Science Center (LA2014233), and all experiments were performed in accordance with the approved guidelines.

FBS, MEM, DMEM, and 100× penicillin and streptomycin mixture were purchased from Gibco (Grand Island, NY, USA). Human BMMSCs and ASCs were cultured in proliferation medium (PM), consisting of 10% (*v*/*v*) FBS, penicillin/streptomycin, and fresh MEM (for hBMMSCs) or DMEM (for hASCs), with 5% CO_2_ atmosphere at 37 °C. The OM comprised fresh DMEM or MEM containing 10 nM dexamethasone, 10 mM β-glycerophosphate, 0.2 mM l-ascorbic acid, 10% (*v*/*v*) FBS, and penicillin/streptomycin. TNF-α was purchased from R&D Systems (Minneapolis, MN, USA), and BAY117082 was purchased from Selleck (Houston, TX, USA). Cells at the fourth to sixth passage were used for the in vitro experiments.

### Preparation of concentrated FFA solution

Flufenamic acid was first dissolved in DMSO to obtain a concentrated solution at 200 mmol L^−1^, to test the effect of FFA at different concentrations on the differentiation of hMSCs, and to choose the optimal concentration for osteogenic differentiation of hMSCs in vitro.

### Cell proliferation assay

Human BMMSCs or ASCs were seeded in 12-well plates at 2 × 10^4^ per well. After that, the cells were cultured in PM, or PM with 25, 50, 100, and 200 μM FFA, respectively. Three wells of cells in the same treatment method were tested daily from day 1 to day 7. The cells were counted using a Cell Counting Kit-8 (CCK8, Dojindo Laboratories, Kumamoto, Japan), according to the manufacturer’s instructions and growth curves of the cells were obtained according to the cell number.

### Alkaline phosphatase staining

Human BMMSCs or ASCs were seeded in 6-well plates at the same initial density. After 7 days of culture in PM, OM, or OM with different concentrations of FFA, the cells were washed with PBS, fixed in 95% cold ethanol, and washed with PBS again. An alkaline phosphatase (ALP) Staining Kit (CWBIO, Beijing, China) was used for staining, according to the manufacturer’s instructions. The cells were then gently washed with distilled water, and images were obtained using a scanner.

### Quantification of ALP activity

Human BMMSCs or ASCs were seeded in 6-well plates at the same initial density. After 7 days of culture in PM, OM, or OM with different concentrations of FFA, the cells were gently washed with PBS first. Then, the cells were collected and centrifuged at 13362×*g* for 30 min at 4 °C after being lysed with 1% Triton X-100 on ice. A BCA protein assay kit (Pierce Thermo Scientific, Waltham, MA, USA) was used to measure the total protein concentration, according to the manufacturer’s instructions. An ALP assay kit (Nanjing Jiancheng Bioengineering Institute, Nanjing, China) was used to measure the ALP activity, and the ALP activity was normalized to the total protein content of each sample.

### Alizarin red S staining and quantification

Human BMMSCs or ASCs were seeded in 6-well plates at the same initial density. After 14 days (hBMMSCs) or 21 days (hASCs) of culture in PM, OM, or OM with different concentrations of FFA, cells were gently washed with PBS, fixed with 95% cold ethanol, and washed with distilled water. Two percent alizarin red S (ARS) staining solution was then used to stain the cells for 20 min at room temperature. After that, the images were scanned.

For alizarin red S quantification, the stained cells were solubilized with 100 mM cetylpyridinium chloride and the OD value of the solution in each well at 562 nm was then measured spectrophotometrically.

### Quantitative real-time reverse transcription PCR

Total RNA was extracted using the TRIzol reagent (Invitrogen, Carlsbad, CA, USA) from hBMMSCs or hASCs cultured in different media for 7 or 14 days, and a Nano Drop 8000 spectrophotometer (Pierce Thermo Scientific) was used to determine the purity and concentration of the total RNA. A PrimeScript RT Reagent Kit (Takara, Tokyo, Japan) was used to perform reverse transcription, according to the manufacturer’s instructions. The mRNA expression of each gene was then tested by qPCR using the SYBR Green Master Mix (Roche Applied Science, Mannheim, Germany) and a 7500 Real-Time PCR Detection System (Applied Biosystems, Foster City, CA, USA) with glyceraldehyde-3-phosphate dehydrogenase (*GAPDH*) as the reference gene. The primer sequences of human *GAPDH*, *ALP*, *RUNX2*, *BGLAP*, *TNF*, *IL6*, *IL8*, and *ICAM1* used for real-time PCR were as follows: *GAPDH*, (forward) 5′-GAAGGTGAAGGTCGGAGTC-3′ and (reverse) 5′-GAAGATGGTGA TGGGATTTC-3′; *ALP*, (forward) 5′-ATGGGATGGGTGTCTCCACA-3′ and (reverse) 5′-CCACGAAGGGGAACTTGTC-3′; *RUNX2*, (forward) 5′-CCGCCTCA GTGATTTAGGGC-3′ and (reverse) 5′-GGGTCTGTAATCTGACTCTGTCC-3′; *BGLAP*, (forward) 5′-CACTCCTCGCCCTATTGGC-3′ and (reverse) 5′-CCCTCCT GCTTGGACACAAAG-3′; *TNF*, (forward) 5′-GCCCAGGCAGTCAGATCATCTT C-3′ and (reverse) 5′-ACAGGCTTGTCACTCGGGGT-3′; *IL6*, (forward) 5′-GCCAG AGCTGTGCAGATGAGT-3′ and (reverse) 5′-AGCA GGCTGGCATTTGTGGT-3′; *IL8*, (forward) 5′-ACCACACTGCGCCAACACAG-3′ and (reverse) 5′-TGCACCCA GTTTTCCTTGGGG-3′; and *ICAM1*, (forward) 5′-TGCACCCAGTTTTCCTTGGG G-3′ and (reverse) 5′-GGCGCCGGAAAGCTGTAGAT-3′.

### Western blot

First, hBMMSCs were washed with cold PBS softly for three times. After that, the cells were lysed in radioimmunoprecipitation assay (RIPA) buffer supplemented with 1% phosphatase inhibitor (Roche) and 2% protease inhibitor cocktail (Roche). After collecting and centrifugation of the cell lysate at 13362*g* at 4 °C for 30 min, supernatants were carefully transferred to new tubes, and the BCA protein assay kit was used to determine the protein concentrations. Thirty-five-microgram total protein of each sample was subjected to 10% SDS-PAGE. Proteins were transferred to a polyvinylidene fluoride membrane (Millipore, Billerica, MA, USA) after electrophoresis. The membrane was then blocked with 5% nonfat milk and then incubated with anti-IκBα (Abcam, Cambridge, UK), anti-p-IKK, anti-p-IκBα, anti-p-P65, anti-P65, anti-RUNX2, or anti-GAPDH (Cell Signaling Technology, Beverly, MA, USA) in TBS at 4 °C overnight. After that, the membrane was washed with Tris-buffered saline-Tween 20 (TBST) buffer for 3 times and then incubated with goat anti-rabbit IgG (Abcam). After that, the membrane was washed with TBST buffer again. Lastly, an ECL Western blot kit (CWBIO) was used to visualize the bands.

### FFA treatment of transplants in vivo

All the mice used in the present study were purchased from Vital River Corporation (Beijing, China) and were maintained in a pathogen-free facility on a 12-h light/dark cycle with water and food provided ad libitum.

For heterotopic bone formation, 6-week-old (*n* = 18) female BALB/C homozygous nude mice were randomly divided into three groups (6 mice per group): PM, PM+DMSO, and PM+50 μM FFA. Next, hBMMSCs for the three groups were cultured in PM, PM with 1‰ DMSO, and PM with 50 μM FFA (FFA was first dissolved in DMSO to get a concentrated solution at 50 mmol L^−1^), respectively, for 10 days. After that, the cells in each treatment group were harvested and incubated with tricalcium phosphate (TCP) carrier (Bicon, Boston, MA, USA) scaffolds at 37 °C for 1 h, followed by centrifugation at 150×*g* for 5 min. The hBMMSCs-scaffolds hybrids were implanted into the dorsal subcutaneous space of the nude mice. The samples were carefully harvested after 8 weeks and analyzed by H&E and Masson staining.

### Ovariectomy and sham operations

Female C57BL6 mice (8 weeks old (*n* = 40)) were randomly divided into two groups. For general anesthesia, pentobarbital sodium (50 mg kg^−1^) was given to the mice by intraperitoneal injections. After that, a bilateral ovariectomy (OVX) or sham operation was performed using standard methods [22].

### In vivo experiment with FFA injection

Four weeks after surgery [23], the 40 OVX or sham mice were randomly divided into four groups of 10 mice for FFA or normal saline (N.S.) injections as follows: (1) Sham mice with N.S.; (2) Sham mice with FFA; (3) OVX mice with N.S.; and (4) OVX with FFA. The in vivo treatment dose was calculated according to the best concentration of promoting osteogenesis in vitro, 50 μM as follows:

An adult mouse (~ 25 g in weight) has a circulating blood volume of ~ 2.5 mL (http://web.jhu.edu/animalcare/procedures/mouse.html). The blood concentration of FFA at 50 μM, the optimal concentration for the in vitro study, was used, and the injection amount of FFA should be ~ 1.4 mg kg^−1^ (the molecular weight of FFA is 281.23 g mol^−1^) [24].

After 1 month of injection of FFA or N.S. with the same volume as the FFA solution per day, the mice were sacrificed and their femurs, hearts, livers, spleens, lungs, and kidneys were carefully collected and fixed in 10% formalin. The blood of each mouse was also carefully collected.

For aged mice, 12-month-old female C57BL6 mice (*n* = 20) were randomly divided into two groups of 10 mice for FFA or N.S. injections. Mice in group one (FFA) were given FFA at 1.4 mg kg^−1^ per day through intraperitoneal injection. Mice in group two (N.S.) were given N.S. in the same volume as the FFA solution. One month later, the mice were sacrificed, and their femurs, hearts, livers, spleens, lungs, and kidneys were carefully collected and fixed in 10% formalin. The blood of each mouse was also carefully collected.

### Analysis of serum biomarker

Serum procollagen I N-terminal propeptide (P1NP) was chosen as the indicator of bone formation. The serum biomarker was measured using an ELISA kit (Cloud-Clone, Katy, TX, USA) according to the manufacturer’s instructions.

### Bone histomorphometry

Bone histomorphometry analysis was performed following a protocol that has been described [25]. To be specific, calcein was given to the mice through intraperitoneal injection at 10 mg kg^−1^ 8 days before they were euthanized for histomorphometry analysis. Seven days after the first injection, calcein was injected again at the same dose. The femurs were carefully collected and fixed in 10% formalin. The bones were then dehydrated, embedded with resin, and sliced for H&E staining. All bone-specific parameters were then measured and calculated.

### Micro-computed tomography and bone morphometric analysis of mice

The femurs were carefully collected and then fixed with 10% formalin for 24 h before being gently washed with 10% sucrose solution. After that, micro-CT images were obtained at a resolution of 8.82 μm, with a tube voltage of 60 kV, a tube current of 500 μA, and an exposure time of 1500 ms. Multimodal 3D visualization software (Inveon Research Workplace; Siemens, Munich, Germany) supplied by the micro-CT system was used for three-dimensional (3D) reconstructions on the basis of two-dimensional (2D) images.

An Inveon Research Workplace (Siemens) was used to calculate the parameters: Bone mineral density (BMD), bone volume/total volume (BV/TV), trabecular thickness (Tb.Th), trabecular number (Tb.N), and trabecular separation (Tb.Sp) in the region of interest (0.5 to 1 mm distal to the proximal epiphysis).

### Statistical analysis

All statistical analyses were performed using SPSS Statistics 20.0 software (IBM). Independent two-tailed Student’s *t* tests were used to analyze the comparisons between two groups. One-way ANOVA and a Tukey’s post hoc test were used to analyze the comparisons between more than two groups. Data shown represents the mean ± standard deviation (SD) of 3 to 10 experiments per group. Values of *p* < 0.05 were considered statistically significant.

## Results

### FFA at low concentrations promoted osteogenic differentiation of hBMMSCs in vitro

**Fig. 1:**
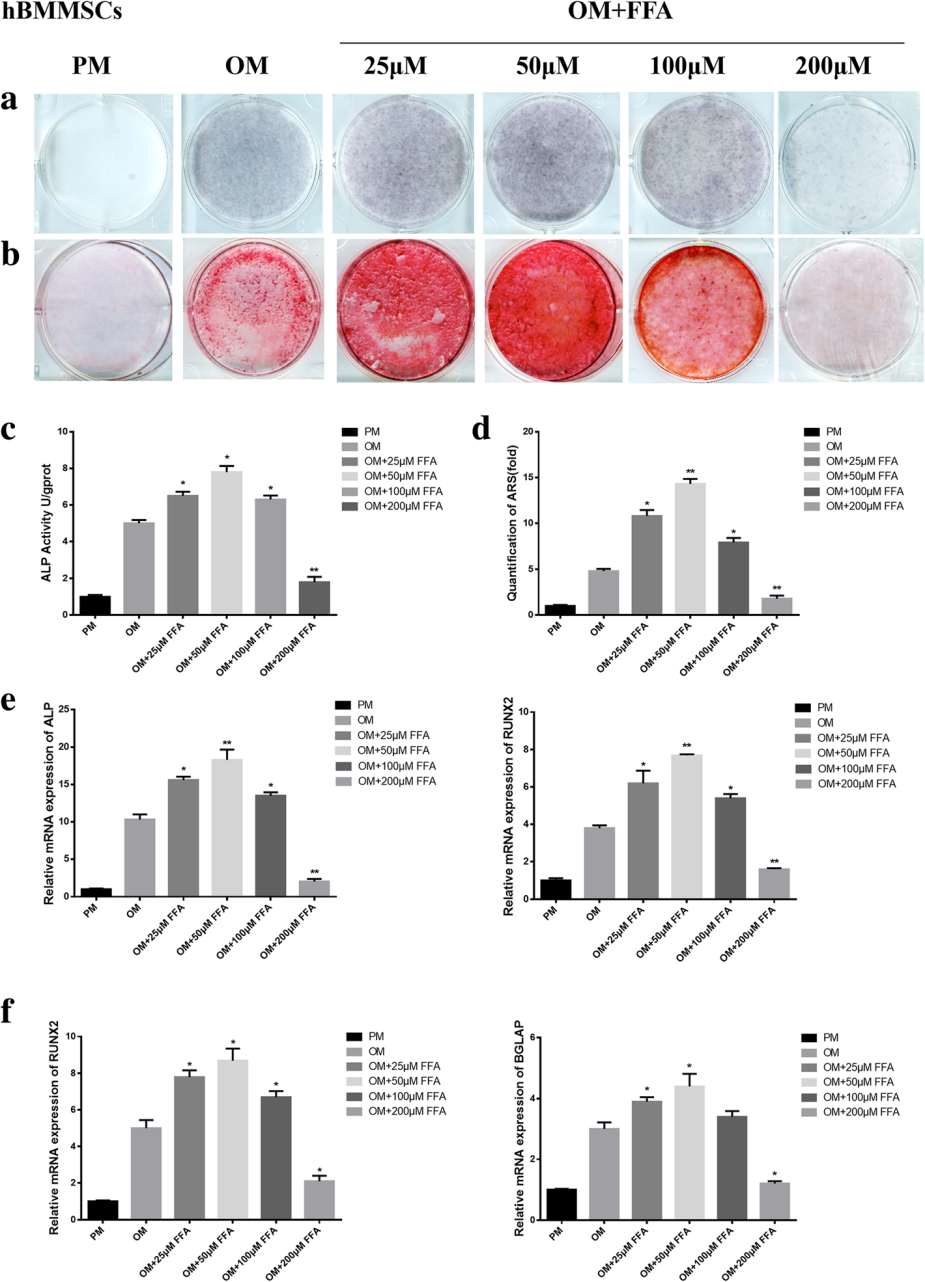
FFA at different concentrations affected osteogenic differentiation of hBMMSCs in vitro*.* **a** 25, 50, and 100 μM FFA increased ALP activity in hBMMSCs, whereas 200 μM FFA decreased ALP activity as shown by ALP staining. Human BMMSCs were cultured in PM, OM, or OM with FFA at 25, 50,100, or 200 μM for 7 days. **b** FFA at 25, 50, and 100 μM accelerated mineralization in hBMMSCs, whereas 200 μM FFA reduced mineralization as shown by ARS staining. Human BMMSCs were cultured in PM, OM, or OM with FFA at 25, 50,100, or 200 μM for 14 days. **c** The result of quantification of ALP activity was consistent with the result of ALP staining. **d** The result of quantification of ARS was consistent with the result of ARS staining. **e** FFA at 25, 50, and 100 μM promoted the expression of *ALP* and *RUNX2* on day 7. However, 200 μM FFA inhibited the expression of the genes mentioned above, as determined by qRT-PCR. **f** FFA at 25, 50, and 100 μM promoted the expression of *RUNX2* and *BGLAP* on day 14, whereas 200 μM FFA inhibited the expression of the genes mentioned above, as determined by qRT-PCR in hBMMSCs. All data are shown as the mean ± SD, *n* = 3. *p < 0.05 and **p < 0.01 compared with OM. FFA, flufenamic acid; hBMMSC, human bone marrow-derived mesenchymal stem cell; PM, proliferation media; OM, osteogenic media; ALP, alkaline phosphatase; ARS, alizarin red S; RUNX2, runt-related transcription factor 2; BGLAP, bone gamma-carboxyglutamate protein; qRT-PCR, quantitative real-time reverse transcription PCR

ALP staining and quantification showed that low concentration FFA promoted the osteogenic differentiation of hBMMSCs cultured in OM on day 7 (Fig. 1a, c). The ARS staining and quantification on day 14 displayed similar results to those of ALP staining and quantification (Fig. 1b, d). FFA at 25, 50, and 100 μM increased the expression level of *ALP*, *RUNX2* (Fig. 1e) at day 7, and *BGLAP* (Fig. 1f) at day 14. However, the promoting effect declined with increasing concentration. The best concentration for osteogenesis promoting was 50 μM. When the concentration reached 200 μM, FFA inhibited osteogenic differentiation (Fig. 1a-f). In addition, the presence of 1‰ DMSO did not have any influence on the osteogenic differentiation of hBMMSCs in vitro (Additional file 1: Figure S1a). Furthermore, 25 to 100 μM FFA did not affect the proliferation of hBMMSCs in vitro, whereas 200 μM FFA inhibited the proliferation of hBMMSCs in vitro (Additional file 2: Figure S2a).

### FFA at low concentrations promoted osteogenic differentiation of hASCs in vitro

**Fig. 2:**
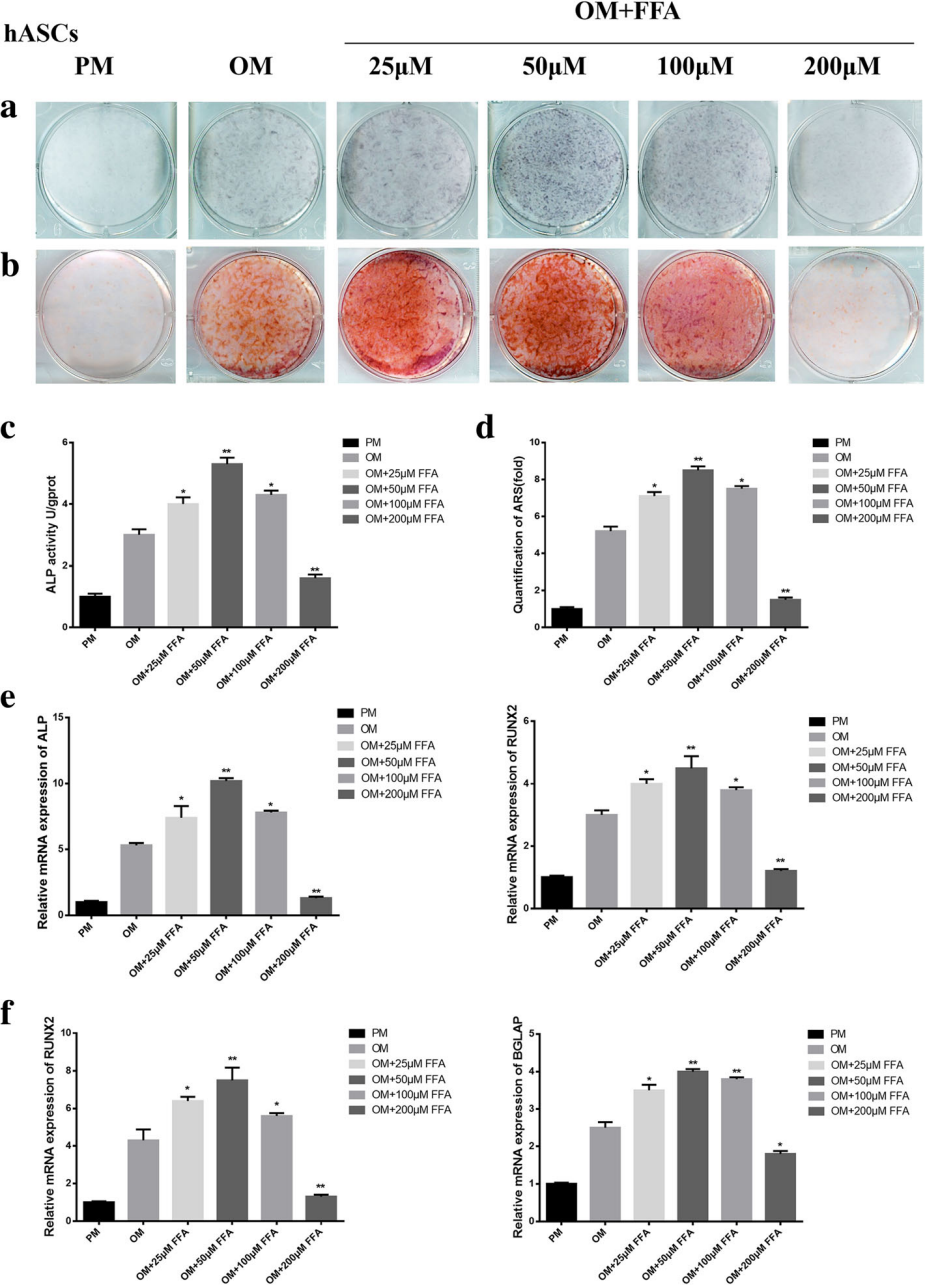
FFA at different concentrations affected osteogenic differentiation of hASCs in vitro*.* **a** 25, 50, and 100 μM FFA increased ALP activity in hASCs, whereas 200 μM FFA decreased ALP activity as shown by ALP staining. Human ASCs were cultured in PM, OM, or OM with FFA at 25, 50,100, or 200 μM for 7 days. **b** FFA at 25, 50, and 100 μM accelerated mineralization in hASCs, whereas 200 μM FFA reduced mineralization as shown by ARS staining. Human ASCs were cultured in PM, OM, or OM with FFA at 25, 50,100, or 200 μM for 14 days. **c** The result of quantification of ALP activity was consistent with the result of ALP staining. **d** The result of quantification of ARS was consistent with the result of ARS staining. **e** FFA at 25, 50, and 100 μM promoted the expression of *ALP* and *RUNX2* on day 7, whereas 200 μM FFA inhibited the expression of the genes mentioned above, as determined by qRT-PCR. **f** FFA at 25, 50, and 100 μM promoted the expression of *RUNX2* and *BGLAP* on day 14, whereas 200 μM FFA inhibited the expression of the genes mentioned above, as determined by qRT-PCR in hASCs. All data are shown as the mean ± SD, *n* = 3. *p < 0.05 and **p < 0.01 compared with OM. FFA, flufenamic acid; hASCs, human adipose-derived stem cell; PM, proliferation media; OM, osteogenic media; ALP, alkaline phosphatase; ARS, alizarin red S; RUNX2, runt-related transcription factor 2; BGLAP, bone gamma-carboxyglutamate protein; qRT-PCR, quantitative real-time reverse transcription PCR

Similar results were obtained in another kind of hMSCs, hASCs. ALP staining and quantification showed that low concentration FFA promoted the osteogenic differentiation of hASCs cultured in OM on day 7 (Fig. 2a, c). The ARS staining and quantification on day 14 displayed similar results to those of ALP staining and quantification (Fig. 2b, d). FFA at 25, 50, and 100 μM increased the expression level of *ALP*, *RUNX2* (Fig. 2e) at day 7, and *BGLAP* (Fig. 2f) at day 14. However, the promoting effect declined with increasing concentration. The best concentration for osteogenesis promoting was 50 μM. When the concentration reached 200 μM, FFA inhibited osteogenic differentiation (Fig. 2a-f). In addition, the presence of 1‰ DMSO did not have any influence on the osteogenic differentiation of hASCs in vitro (Additional file 1: Figure S1b). Furthermore, 25 to 100 μM FFA did not affect the proliferation of hASCs in vitro, whereas 200 μM FFA inhibited the proliferation of hASCs in vitro. (Additional file 2: Figure S2b).

### Low concentration FFA enhanced osteogenic differentiation of hBMMSCs in vivo

**Fig. 3:**
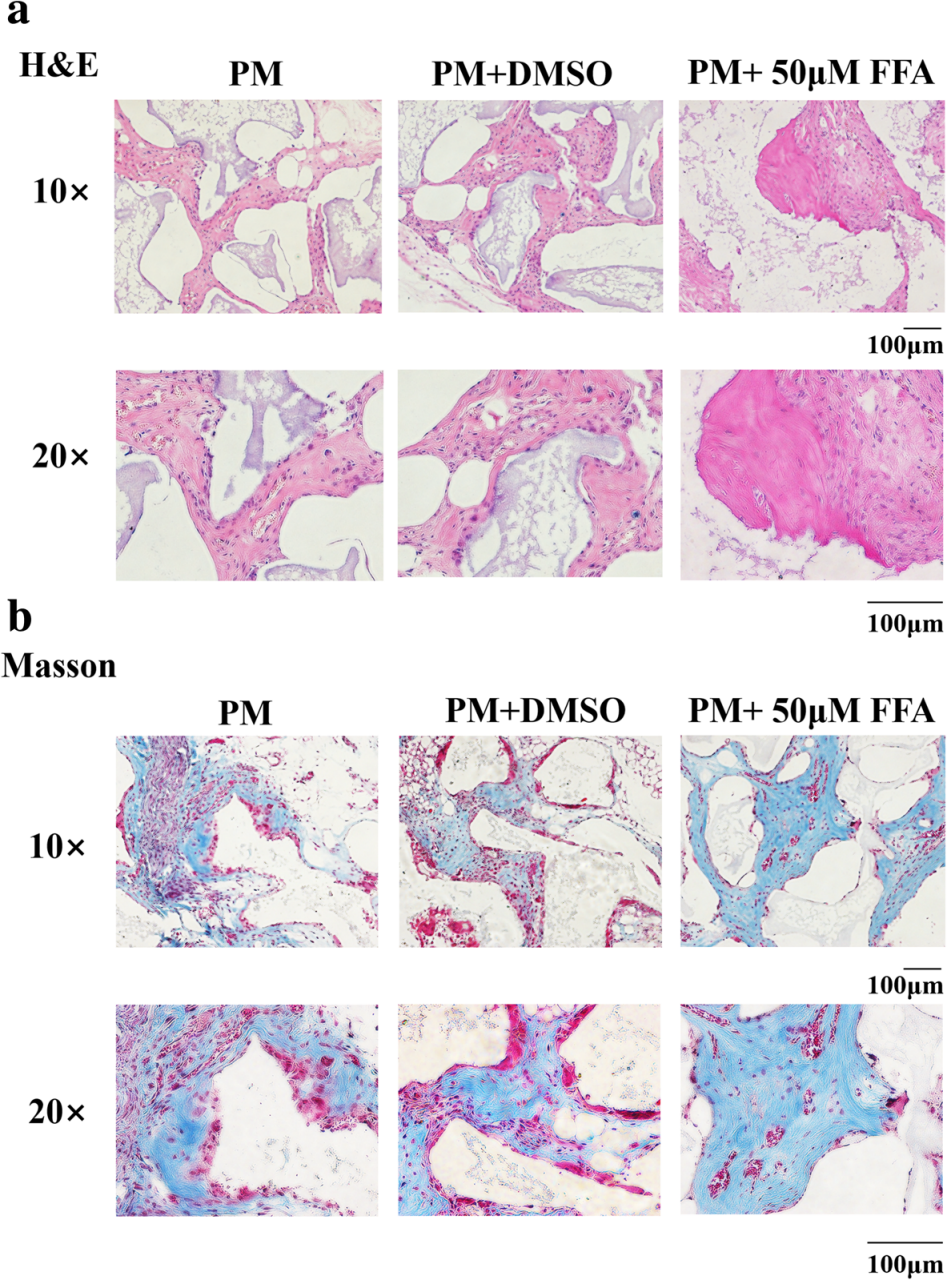
FFA at 50 μM promoted osteogenic differentiation of hBMMSCs in vivo. **a** H&E staining of PM, PM+DMSO, and PM+FFA groups. **b** Masson’s staining of PM, PM+DMSO, and PM+FFA groups. PM, proliferation media; FFA, flufenamic acid; hBMMSC, human bone marrow-derived mesenchymal stem cell; H&E, hematoxylin and eosin

According to the in vitro experiments, 50 μM FFA had the best promoting effect on osteogenesis. So, 50 μM FFA was used in the following in vivo experiments. Human [BMMSCs] were treated in the following three ways: PM, PM with 1‰DMSO, and PM with 50 μM FFA for 10 days. (The treated cells) were loaded onto TCP carrier scaffolds and implanted in the dorsal subcutaneous space of nude mice (six mice per group). We collected the implantation samples after 8 weeks and performed the analysis. H&E staining revealed more newly formed bone in the PM+FFA group than in the other two groups (Fig. 3a). Masson’s trichrome staining also showed that there was more collagen organization (blue color) in the PM+FFA group than in the other two groups (Fig. 3b). There were no significant differences between the PM group and PM+DMSO group, which means the presence of 1‰ DMSO did not influence the osteogenic differentiation of hBMMSCs in vivo.

### Low concentration FFA suppressed bone loss in OVX mice and partially reversed bone loss in aged mice

**Fig. 4:**
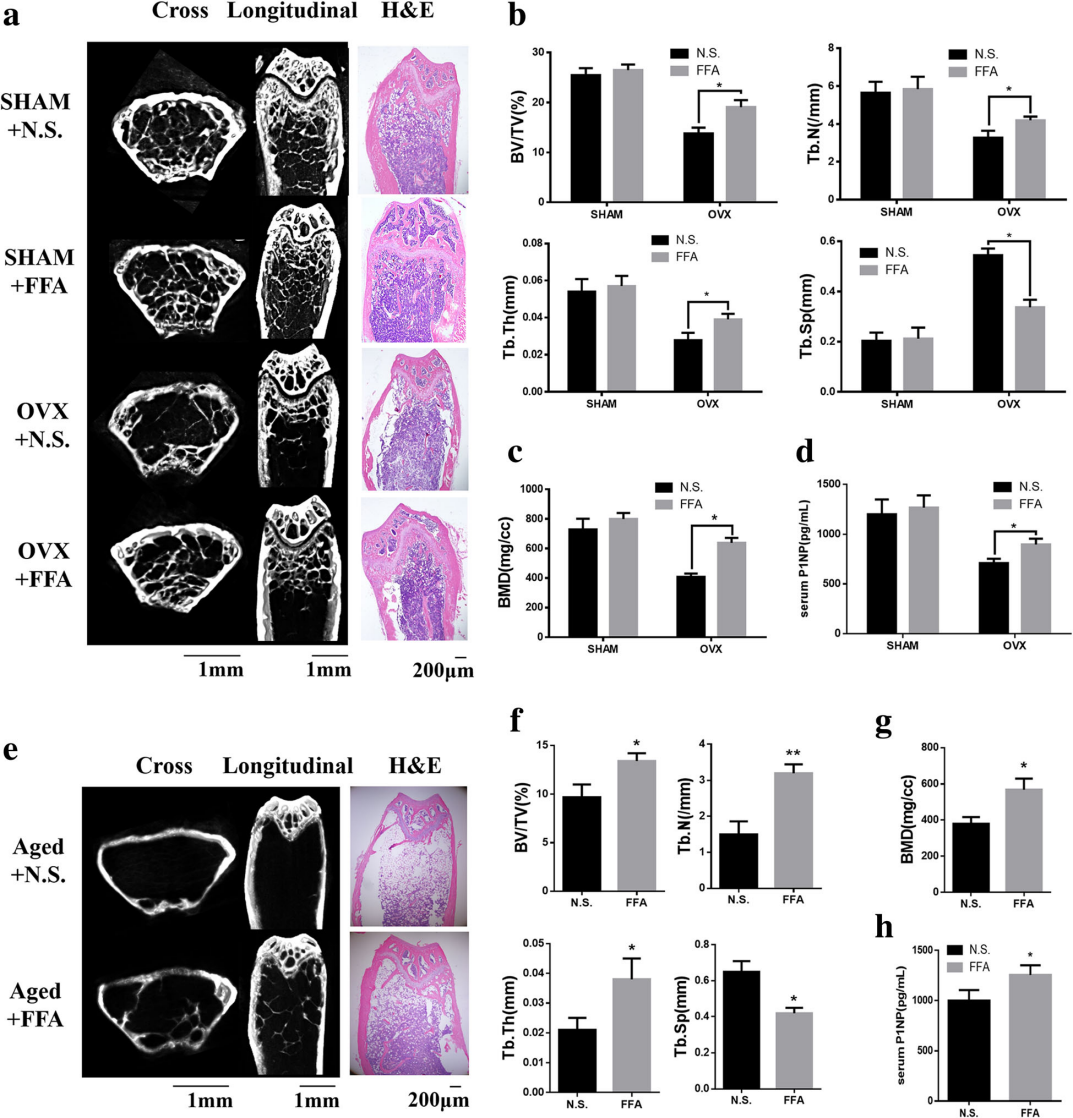
FFA at 50 μM suppressed osteoporosis in OVX and partially reversed bone loss in aged mice. **a** Micro-CT and H&E staining images of femurs in SHAM mice with N.S. treatment, SHAM mice with FFA treatment, OVX mice with N.S. treatment, and OVX mice with FFA treatment for 4 weeks. **b** Compared with the OVX mice with N.S. treatment, the OVX mice treated with FFA showed improvement in bone volume, trabecular number, and trabecular thickness and a reduction in trabecular separation. **c** Compared with the OVX mice with N.S. treatment, the OVX mice treated with FFA showed improvement in BMD. **d** FFA increased serum bone formation marker, P1NP in OVX mice. **e** Micro-CT and H&E staining images of femurs in aged mice treated with N.S. and aged mice with FFA treatment for 4 weeks. Images of femurs in aged mice with N.S. and aged mice with FFA treatment for 4 weeks. **f** Aged mice treated with FFA had improvement of bone volume, trabecular number, and trabecular thickness and a reduction in trabecular separation compared with aged mice with N.S. treatment. **g** Aged mice treated with FFA had improvement of BMD. **h** FFA increased serum bone formation marker, P1NP in aged mice. Scale bars for μCT images are 1 mm. Scale bars for H&E staining images are 200 μm. All data are shown as the mean ± SD. *p < 0.05 compared with OVX mice with N.S. treatment (b-d). *p < 0.05 and **p < 0.01 compared with aged mice treated with N.S. (f-h). FFA, flufenamic acid; OVX, ovariectomized; BMD, bone mineral density; micro-CT, micro-computed tomography; N.S., normal saline

To further demonstrate the clinical relevance of our results, whether FFA could suppress osteoporosis caused by ovariotomy and aging in mice was investigated. Forty 6-week-old female mice were divided into four groups of 10 mice: SHAM, SHAM+FFA, OVX, and OVX+FFA. No significant difference was found between SHAM mice with N.S. treatment and SHAM mice with FFA treatment in micro-CT and H&E staining of the femurs (Fig. 4a), whereas FFA treatment partially blocked trabecular bone loss in the OVX mice. Moreover, in the OVX mice, there was a decrease in bone volume (Fig. 4b), trabecular number (Fig. 4b), trabecular thickness (Fig. 4b), and BMD (Fig. 4c) and an increase in trabecular separation (Fig. 4b), while treatment of FFA improved these parameters in the OVX mice, which were confirmed by bone histomorphometry analysis (Table 1). What is more, FFA treatment increased the bone formation marker P1NP (Fig. 4d) in serum of OVX mice.

**Table 1:** Bone histomorphometry analysis of SHAM and OVX mice

	SHAM+N.S.	SHAM+FFA	OVX+N.S.	OVX+FFA
BV/TV (%)	11.070 ± 1.87	12.532 ± 1.92	8.132 ± 1.22	9.370 ± 1.10*
Tb.Ar (%)	5.315 ± 0.42	5.494 ± 0.76	3.570 ± 0.52	4.088 ± 0.79
Tb.Th (μm)	11.874 ± 1.35	13.773 ± 2.07*	7.385 ± 0.86	8.044 ± 1.06
Tb.Sp (μm)	267.538 ± 38.95	255.379 ± 27.66	329.873 ± 37.922	295.552 ± 26.816*
Tb.N (#/mm)	3.95 ± 0.46	4.23 ± 0.59	1.96 ± 0.35	2.31 ± 0.29*
MAR (μm/day)	1.668 ± 0.33	1.709 ± 0.29	1.241 ± 0.25	1.533 ± 0.31*
BFR (μm/day)	21.872 ± 1.97	20.511 ± 2.85	13.077 ± 1.97	14.472 ± 3.69
Ob.S/BS (%)	10.010 ± 1.86	11.677 ± 1.98	8.406 ± 1.14	9.002 ± 1.37
N.Ob/T.Ar	81.624 ± 19.75	92.331 ± 10.948	58.376 ± 7.83	67.679 ± 9.03*
N.Ob/B.Pm	13.978 ± 2.912	13.470 ± 4.011	7.870 ± 1.90	9.010 ± 1.65

*p < 0.05, **p < 0.01, SHAM+FFA compared with SHAM+N.S., OVX+FFA compared with OVX + N.S

Micro-CT and H&E staining also showed that trabecular bone loss was partially reversed by FFA in aged mice (Fig. 4e). Specifically, compared with the aged mice treated with N.S., we observed an increase in bone volume (Fig. 4f), trabecular number (Fig. 4f), trabecular thickness (Fig. 4f), and BMD (Fig. 4g) and a reduction in trabecular separation (Fig. 4f) in the aged mice treated with FFA, which were confirmed by bone histomorphometry analysis (Table 2). What is more, FFA treatment increased the bone formation marker P1NP (Fig. 4h) in serum of aged mice.

**Table 2:** Bone histomorphometry analysis of aged mice

	Aged+N.S.	Aged+FFA
BV/TV (%)	3.012 ± 0.46	4.035 ± 0.39***
Tb.Ar (%)	2.615 ± 0.32	3.498 ± 0.66**
Tb.Th (μm)	3.874 ± 0.45	4.373 ± 0.57*
Tb.Sp (μm)	589.783 ± 66.01	525.615 ± 58.19*
Tb.N (#/mm)	1.05 ± 0.26	1.61 ± 0.39**
MAR (μm/day)	0.893 ± 0.13	1.009 ± 0.24
BFR (μm/day)	12.364 ± 1.86	13.196 ± 2.35
Ob.S/BS (%)	6.007 ± 0.73	6.462 ± 1.04
N.Ob/T.Ar	47.531 ± 11.03	55.198 ± 9.038
N.Ob/B.Pm	7.428 ± 1.361	8.598 ± 1.023*

*p < 0.05, **p < 0.01, ***p < 0.001

Besides, FFA at the concentration used in the present study did not cause toxicity or inflammatory changes in the, kidney, liver, spleen, lung, and heart of the SHAM and aged mice (Additional file 3: Figure S3).

FFA at low concentrations promoted the osteogenic differentiation of hMSCs by inhibiting the NF-κB pathway.

**Fig. 5:**
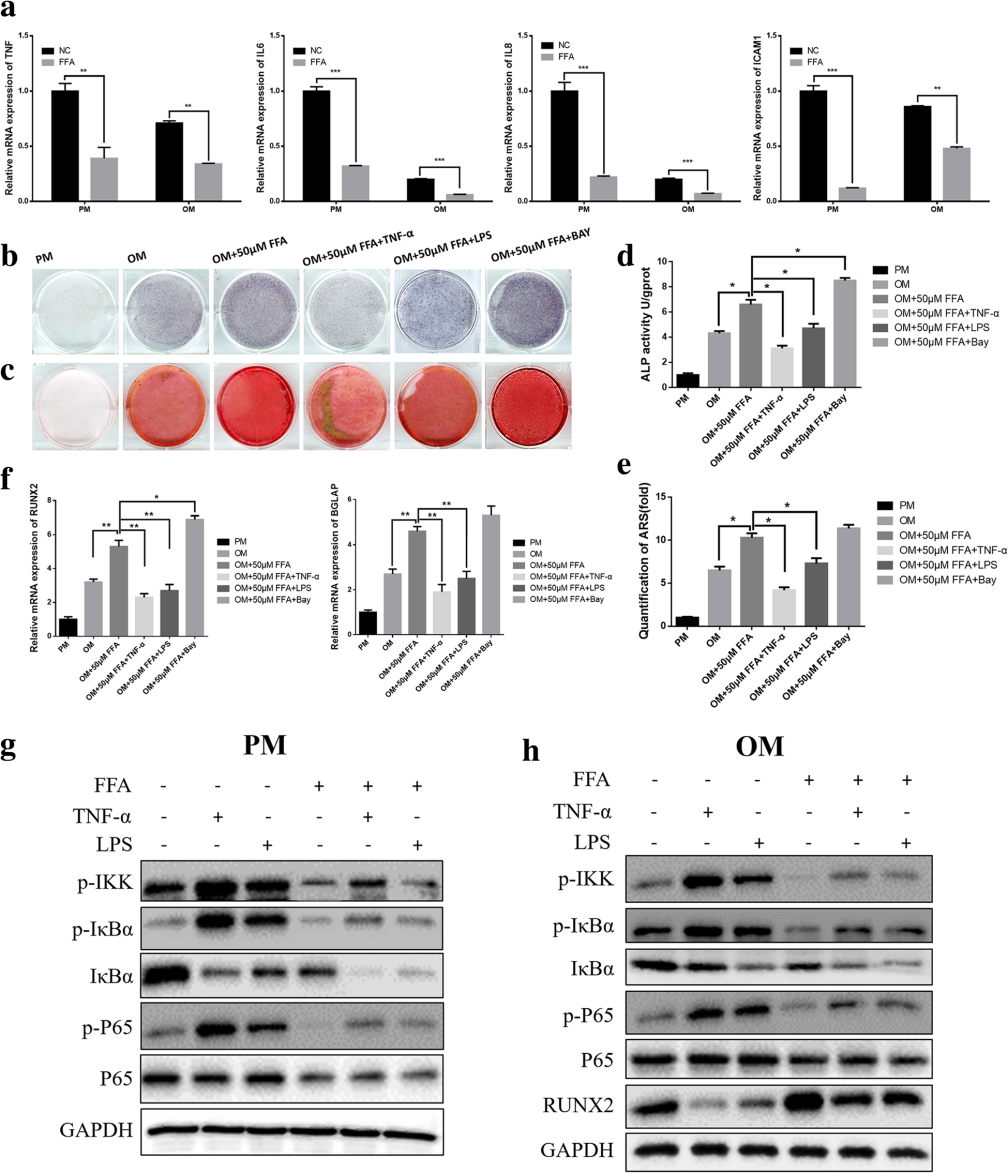
FFA in low concentrations promoted the osteogenic differentiation of hMSCs by inhibiting the NF-κB pathway. **a** FFA at 50 μM downregulated the expression of *TNF*, *IL6*, *IL8*, and *ICAM1* both in PM and OM in hBMMSCs, as determined by qRT-PCR. **b** FFA at 50 μM could not increase ALP activity in hBMMSCs in the presence of TNF-α or LPS. Yet FFA at 50 μM increased ALP activity more in hBMMSCs in the presence of Bay117082. Human BMMSCs were treated with PM, OM, OM with FFA at 50 μM, or OM with FFA at 50 μM and TNF-α or LPS or Bay117082 for 7 days before ALP staining. **c** FFA at 50 μM no longer accelerated mineralization in hBMMSCs in the presence of TNF-α or LPS. Yet FFA at 50 μM accelerated mineralization more in hBMMSCs in the presence of Bay117082. Cells were treated with PM, OM, OM with FFA at 50 μM, or OM with FFA at 50 μM and TNF-α or LPS or Bay117082 for 14 days, and calcium deposition was then tested using ARS staining. **d** The result of quantification of ALP activity was consistent with the result of ALP staining. **e** The result of quantification of ARS was consistent with the result of ARS staining. **f** TNF-α and LPS downregulated the expression of *RUNX2* and *BGLAP* in hBMMSCs, which had been increased by 50 μM FFA, as determined by qRT-PCR. Bay117082 further upregulated the expression of *RUNX2* and *BGLAP* in hBMMSCs, which had been increased by 50 μM FFA, as determined by qRT-PCR. **g** Western blot of protein expression of p-IKK, p-IκBα, IκBα, p-P65, P65, and the internal control GAPDH. Human BMMSCs were cultured in PM or PM with FFA for 7 days before treated with TNF-α or LPS for 30 min. **h** Western blot of protein expression of p-IKK, p-IκBα, IκBα, p-P65, P65, RUNX2, and the internal control GAPDH. Human BMMSCs were cultured in OM or OM with FFA for 7 days before treated with TNF-α or LPS for 30 min. All data are shown as the mean ± SD, *n* = 3. *p < 0.05, **p < 0.01, and ***p < 0.001. FFA, flufenamic acid; hBMMSC, human bone marrow-derived mesenchymal stem cell; PM, proliferation media; OM, osteogenic media; ALP, alkaline phosphatase; ARS, alizarin red S; RUNX2, runt-related transcription factor 2; BGLAP, bone gamma-carboxyglutamate protein; qRT-PCR, quantitative real-time reverse transcription PCR; hMSC, human mesenchymal stem cell; NF-κB, nuclear factor kappa B; TNF-α, tumor necrosis factor alpha

Next, to explore the underlying mechanisms how FFA regulates osteogenic differentiation, cells treated with 50 μM FFA in PM and OM were subjected to mRNA analysis for several key regulators of osteogenesis. To our surprise, in the pretest, we first found that 50 μM FFA significantly attenuated the expression of the target genes of NF-κB, either in hBMMSCs cultured in PM or OM for 7 days (Fig. 5a). Based on this, we supposed the NF-κB signaling pathway was downregulated by FFA and that FFA at low concentrations promoted osteogenesis by inhibiting the NF-κB pathway. Therefore, we added the activators of NF-κB signaling pathway, TNF-α, and LPS to OM with 50 μM FFA. ALP staining and quantification showed that 50 μM FFA no longer promoted osteogenesis of hBMMSCs in the presence of TNF-α or LPS (Fig. 5b, d). Meanwhile, the inhibitor of NF-κB signaling, Bay117082, enhanced the promoting effect of FFA on osteogenesis. The ARS staining and quantification on day 14 displayed results similar to those of ALP staining and quantification (Fig. 5c, e). Compared with OM with 50 μM FFA, TNF-α and LPS significantly attenuated the expression level of *RUNX2* (Fig. 5f) at day 7 and *BGLAP* (Fig. 5f) at day 14, whereas Bay117082 upregulated the expression. To further confirm the specific effect of FFA on NF-κB signaling pathway, we tested the effect of FFA on the key proteins of NF-κB signaling pathway by western blot. Human BMMSCs were cultured in PM or PM with 50 μM FFA for 7 days and then treated with TNF-α or LPS for 30 min. The results showed that in PM condition, TNF-α and LPS activated the phosphorylation of IKK and IκBα in 30 min, while the total IκBα was reduced. FFA treatment blocked the phosphorylation of IKK and IκBα as well as decreased total IκBα (Fig. 5g). Besides, FFA also blocked the phosphorylation of P65 activated by TNF-α and LPS and slightly decreased total P65 (Fig. 5 g). In addition, hBMMSCs were cultured in OM or OM with 50 μM FFA for 7 days and then treated with TNF-α or LPS for 30 min. In OM condition, FFA treatment not only had the same effect on the proteins tested in PM, but also increased the expression of RUNX2 remarkably (Fig. 5h).

## Discussion

In this study, we found that FFA at low concentrations could promote osteogenic differentiation of hMSCs both in vitro and in vivo. We also observed that the effect declined as the concentration of FFA increased. In addition, the best concentration to promote osteogenesis was 50 μM. These results suggested that FFA at low concentrations might promote hMSC differentiation toward the osteoblastic lineage. Most importantly, we discovered that FFA not only promoted osteogenic differentiation effectively, but also suppressed bone loss in OVX mice and aged mice. Mechanistically, FFA promotes osteogenic differentiation by inhibiting the NF-κB signaling pathway. The present study provides valuable clues for a new use of FFA to treat bone metabolic diseases, such as osteoporosis.

The in vitro experiments in the present study revealed that FFA could affect the differentiation of hMSCs. Little evidence of the promoting effect of FFA at low concentrations on osteogenic differentiation of hMSCs was available in previous studies, except for a clinical case report which showed that chronic use of niflumic acid, a member of the fenamic acids, might lead to osteosclerosis [26]. This finding was strengthened by the promoting effect of 50 μM FFA on the osteogenic differentiation of hMSCs in vivo and supported by the inhibiting effect of FFA on bone loss in OVX mice and aged mice. Taken together, this evidence indicated that FFA could play a role in promoting osteogenic differentiation of hMSCs and bone metabolism.

Several studies have focused on the effects of different NSAIDs on bones and osteocytes, including a series of biological behaviors such as adhesion, proliferation, and differentiation [27–32]. However, the effect differs with the change in drug and concentration. Perhaps, this is because the members of NSAIDs are quite different in their chemical structures and other properties, which may cause different effects, apart from their expected anti-inflammatory and analgesic behaviors. Currently, it is clear that aspirin can promote osteogenesis of MSCs and attenuate bone loss [10, 33–35]. However, the effective concentration is about 0.25–1.5 mmol according to previous studies, which is far higher than the effective concentration of FFA. As the representative of fenamic acids, FFA is approved by US Food and Drug Administration (FDA) to treat several diseases in the clinic, such as rheumatic arthritis [36, 37]. FFA is inexpensive and can be obtained easily. Thus, our study provides clues for the application of FFA as a new, safe, and economical choice for osteoporosis treatment.

Our study revealed that FFA promoted osteogenesis of hMSCs by inhibition of the NF-κB signaling pathway. NF-κB signaling pathways are associated with bone metabolism [14–19]. In addition, previous studies have shown that FFA can inhibit the NF-κB signaling pathway [38]. Our findings are in agreement with the previous studies. However, this study is the first to point out that FFA promotes osteogenesis through the NF-κB signaling pathway. Although the mechanism of action by which FFA promotes osteogenesis of hMSCs has not been completely elucidated, it is mediated, at least partly, by inhibition of NF-κB signaling. The proliferation curves of the cells showed that 200 μM FFA inhibited the proliferation of hMSCs, which might partially account for the inhibiting effect of high concentration FFA on osteogenic differentiation of hMSCs. Besides, high dose of drugs may cause varied side effects, which means less value in clinic. Thus, we did not deeply study the mechanism how FFA at high concentration inhibited osteogenic differentiation of hMSCs.

However, our study still has several limitations. We only evaluated the signaling pathways by which FFA at low concentrations promoted osteogenesis of hMSCs and partially explained why high concentration FFA inhibited osteogenesis of hMSCs. The complete reason remains unclear. Besides, according to our results, it was clear that the best concentration to promote osteogenesis was between 25 and 100 μM, and among the chosen concentrations in the present study, 50 μM FFA had the best promoting effect. However, some concentrations between 25 and 100 μM might have better effect than 50 μM and further studies are needed to determine the optimal concentration. Moreover, to promote its clinical transformation, further evaluation of the specific treatment concentration in humans is required.

Overall, our results showed that FFA at low concentrations could promote osteogenic differentiation of hMSCs, both in vitro and in vivo, by inhibition of NF-κB signaling. It could also suppress bone loss caused by ovariotomy and aging in mice, suggesting that FFA has a potential therapeutic value to treat osteoporosis.

## Conclusion

FFA in low concentration promotes osteogenic differentiation of hMSCs in vitro, and the effect declines with increasing concentration. The best concentration for osteogenesis promoting is 50 μM. Fifty micromolar FFA can also promote osteogenic differentiation of hMSCs in vivo. Besides, 50 μM FFA suppress bone loss in OVX mice and aged mice. Mechanistically, FFA promotes osteogenesis through inhibiting NF-κB signaling pathway. Collectively, our study suggested that FFA could be used in bone tissue engineering or osteoporosis by promoting osteogenic differentiation of human MSCs.

## Additional files

Additional file 1: **Figure S1.** DMSO (1‰) did not affect the osteogenic differentiation of hMSCs. a 1‰ DMSO did not affect ALP activity in hBMMSCs as tested by ALP staining. b 1‰ DMSO did not affect ALP activity in hASCs as tested by ALP staining. ALP, alkaline phosphatase; hMSC, human mesenchymal stem cell; hBMMSC, human bone marrow-derived mesenchymal stem cell; hASC, human adipose derived stem cell. (TIF 744 kb)

Additional file 2: **Figure S2.** High concentration FFA inhibited proliferation of hMSCs. a 200 μM FFA inhibited proliferation of hBMMSCs whereas 25, 50, and 100 μM FFA caused no significant differences in the proliferative capacities of the cells compared with no FFA treatment during day 1 (1) to day 7 (7), as shown by the growth curve of cells. b 200 μM FFA inhibited proliferation of hASCs whereas 25, 50, and 100 μM FFA caused no significant differences in the proliferative capacities of the cells compared with no FFA treatment during day 1 (1) to day 7 (7), as shown by the growth curve of cells. FFA, flufenamic acid; hMSC, human mesenchymal stem cell; hASC, human adipose derived stem cell; hBMMSC, human bone marrow-derived mesenchymal stem cell. (TIF 373 kb)

Additional file 3: **Figure S3.** FFA caused no toxicity or inflammation in the viscera of mice. Intraperitoneal injection of FFA for 1 month did not cause toxicity or inflammation changes in the kidney, liver, spleen, lung, or heart of SHAM and aged mice. Scale bar = 500 μm. FFA, flufenamic acid. (TIF 11408 kb)

## Data Availability

The authors confirm that all data underlying the findings are fully available.

## Abbreviations

ALP: Alkaline phosphatase
ARS: Alizarin red S
BGLAP: Bone gamma-carboxyglutamic acid-containing protein
BMD: Bone mineral density
BMMSCs: Bone marrow mesenchymal stem cells
BV/TV: Trabecular bone volume/tissue volume
DMSO: Dimethyl sulfoxide
ELISA: Enzyme-linked immunosorbent assay
FFA: Flufenamic acid
GAPDH: Glyceraldehyde 3-phosphate dehydrogenase
H&E: Hematoxylin and eosin
hASCs: Human adipose-derived stem cells
ICAM1: Intercellular adhesion molecule 1
IL: Interleukin
LPS: Lipopolysaccharide
Micro-CT: Micro-computed tomography
MSCs: Mesenchymal stem cells
NF-κB: Nuclear factor-κB
NSAIDs: Non-steroidal anti-inflammatory drugs
OCN: Osteocalcin
OM: Osteogenic medium
P1NP: Procollagen I N-terminal propeptide
PM: Proliferation medium
qRT-PCR: Quantitative reverse transcription PCR
RUNX2: Runt-related transcription factor 2
Tb.N: Trabecular number
Tb.Sp: Trabecular spacing
Tb.Th: Trabecular thickness
TNF: Tumor necrosis factor
