# Supplementary figures and images for: Low concentration flufenamic acid enhances osteogenic differentiation of mesenchymal stem cells and suppresses bone loss by inhibition of the NF-κB signaling pathway
Source: Stem Cell Res Ther. 2019 Jul 19;10:213. doi: 10.1186/s13287-019-1321-y (PMC6642517; doi:10.1186/s13287-019-1321-y)

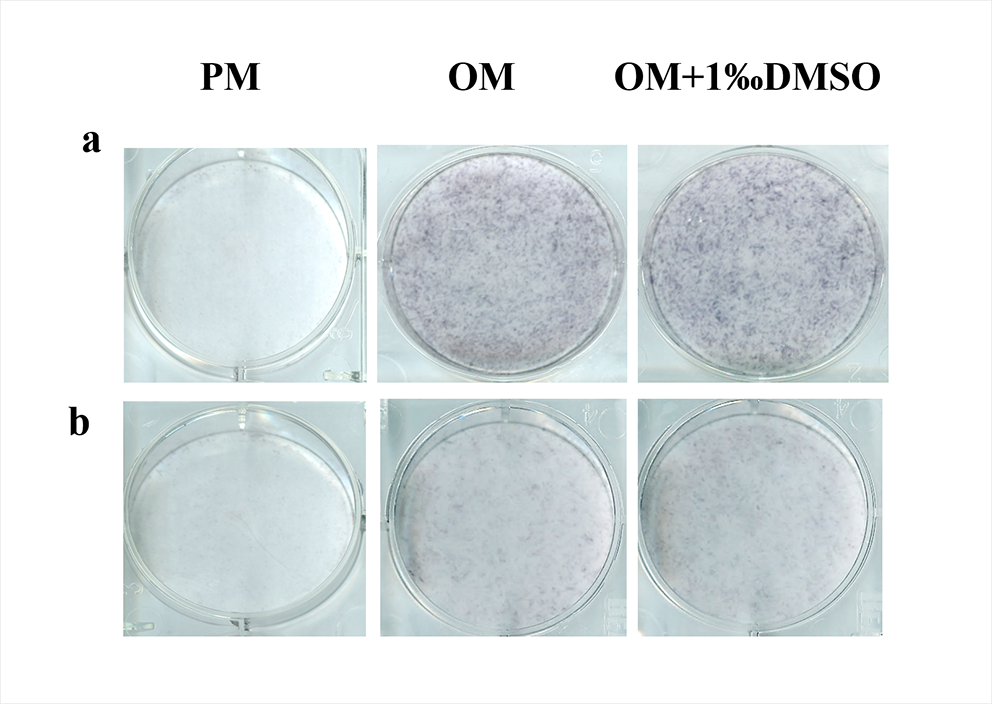

Supplement: Supplementary file 1 — Figure S1. DMSO (1‰) did not affect the osteogenic differentiation of hMSCs. a 1‰ DMSO did not affect ALP activity in hBMMSCs as tested by ALP staining. b 1‰ DMSO did not affect ALP activity in hASCs as tested by ALP staining. ALP, alkaline phosphatase; hMSC, human mesenchymal stem cell; hBMMSC, human bone marrow-derived mesenchymal stem cell; hASC, human adipose derived stem cell. (TIF 744 kb) [file 13287_2019_1321_MOESM1_ESM.tif]

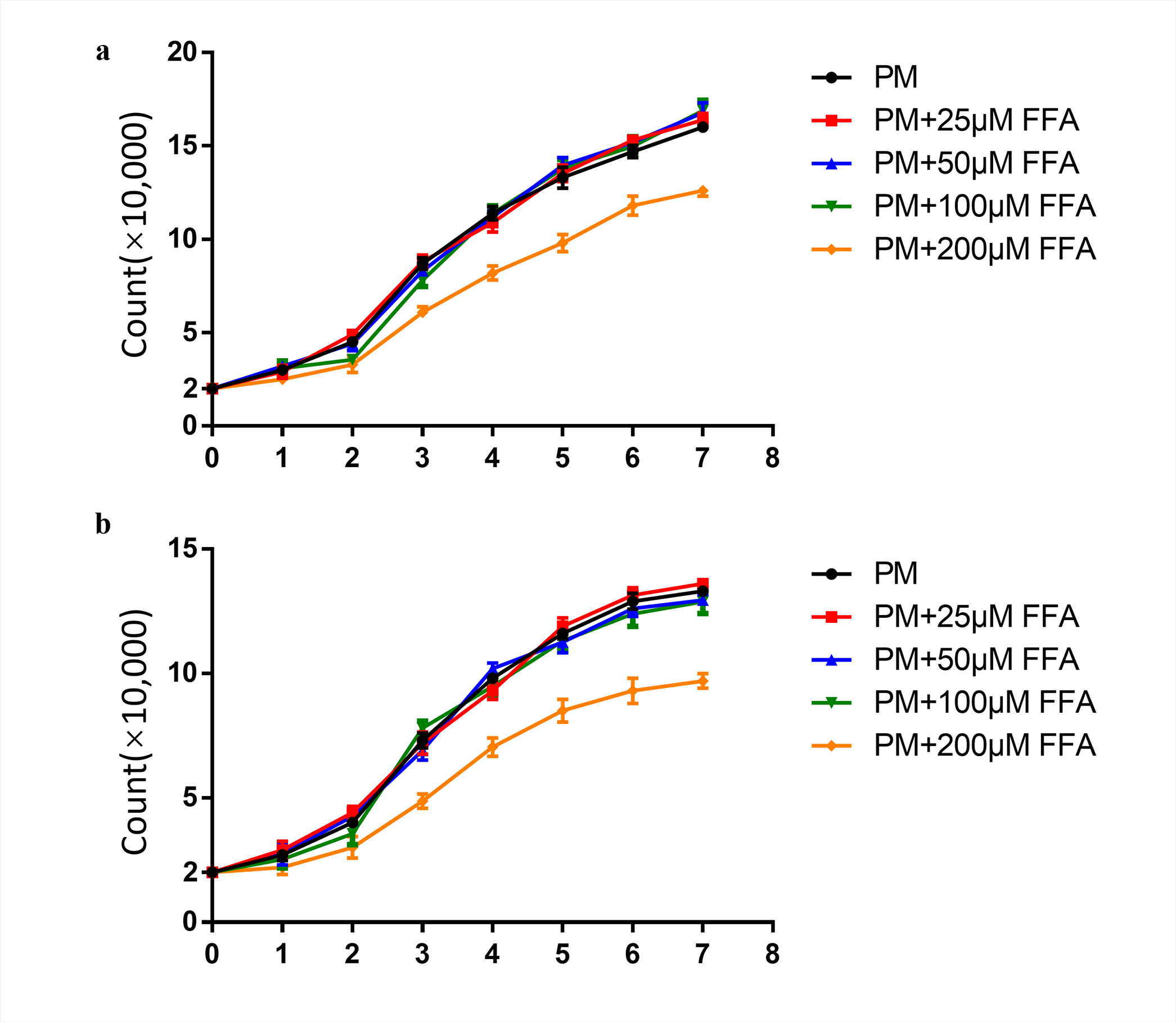

Supplement: Supplementary file 2 — Figure S2. High concentration FFA inhibited proliferation of hMSCs. a 200 μM FFA inhibited proliferation of hBMMSCs whereas 25, 50, and 100 μM FFA caused no significant differences in the proliferative capacities of the cells compared with no FFA treatment during day 1 (1) to day 7 (7), as shown by the growth curve of cells. b 200 μM FFA inhibited proliferation of hASCs whereas 25, 50, and 100 μM FFA caused no significant differences in the proliferative capacities of the cells compared with no FFA treatment during day 1 (1) to day 7 (7), as shown by the growth curve of cells. FFA, flufenamic acid; hMSC, human mesenchymal stem cell; hASC, human adipose derived stem cell; hBMMSC, human bone marrow-derived mesenchymal stem cell. (TIF 373 kb) [file 13287_2019_1321_MOESM2_ESM.tif]

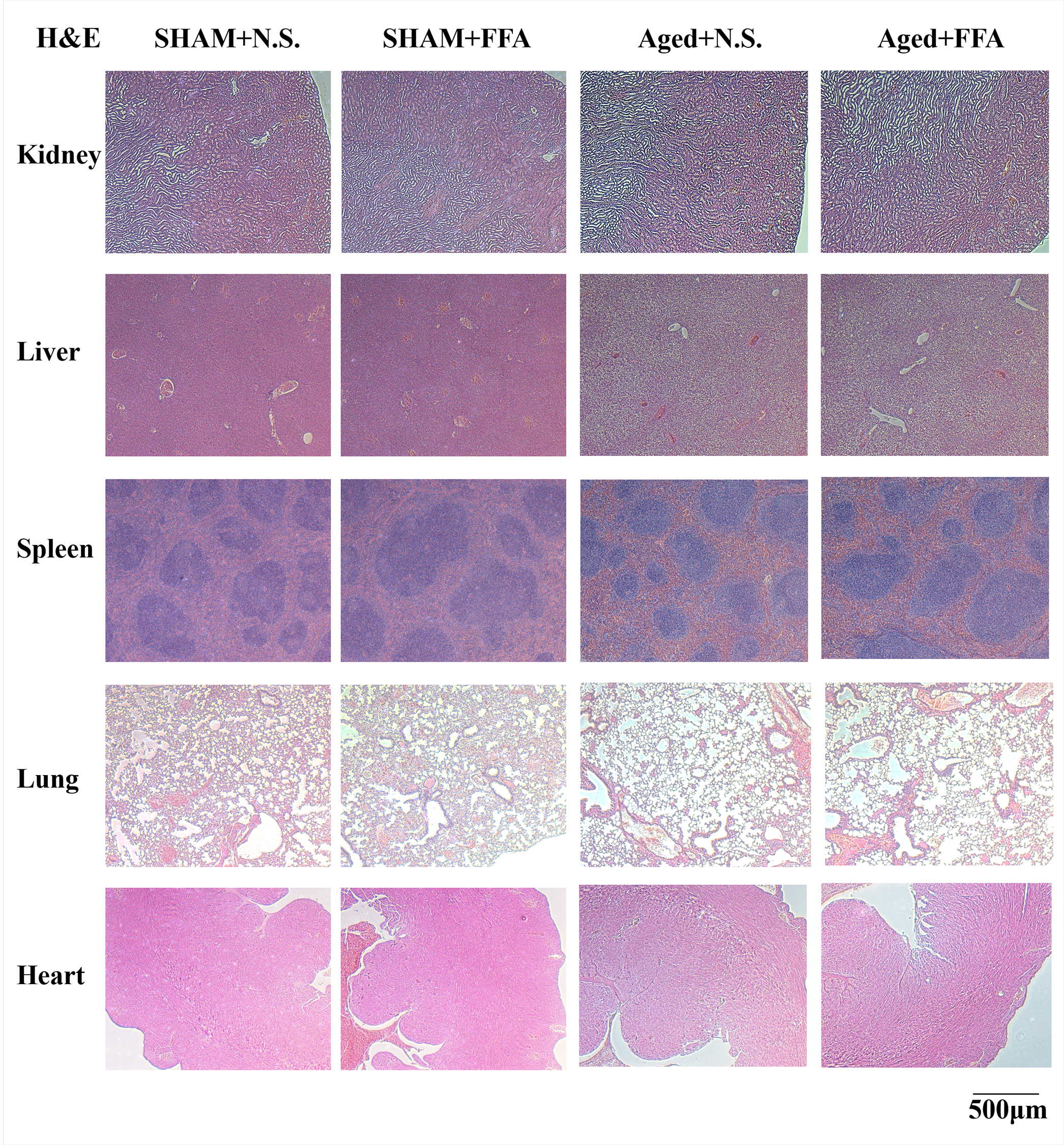

Supplement: Supplementary file 3 — Figure S3. FFA caused no toxicity or inflammation in the viscera of mice. Intraperitoneal injection of FFA for 1 month did not cause toxicity or inflammation changes in the kidney, liver, spleen, lung, or heart of SHAM and aged mice. Scale bar = 500 μm. FFA, flufenamic acid. (TIF 11408 kb) [file 13287_2019_1321_MOESM3_ESM.tif]
